# Modeling the Thermal Conductivity Inhomogeneities of Injection-Molded Particle-Filled Composites, Caused by Segregation

**DOI:** 10.3390/polym11101691

**Published:** 2019-10-16

**Authors:** András Suplicz, Orsolya Viktória Semperger, József Gábor Kovács

**Affiliations:** Department of Polymer Engineering, Faculty of Mechanical Engineering, Budapest University of Technology and Economics, Műegyetem rkp. 3, H- 1111 Budapest, Hungary; suplicz@pt.bme.hu (A.S.); sempergero@pt.bme.hu (O.V.S.)

**Keywords:** Segregation of fillers, injection-molded composites, effective thermal conductivity, polymer-matrix composites, thermal conductivity modeling

## Abstract

Many applications require new materials that have good thermal conductivity, are electrical insulators and can be processed easily and with relatively little energy. A new innovative solution for this problem is thermally conductive composites, which can replace metals in many cases. Many papers have focused on the prediction of their thermal conductivity. At the same time segregation has to be taken into account in the case of composites because it affects the distribution of thermally conductive particles, and thus local thermal conductivities. In this paper, we examined and modeled segregation during injection molding and its effect on thermal conductivity. We injection-molded samples from polypropylene with glass beads of different sizes and analyzed their filler content as a function of the flow path. We described the distribution of the filler with a mathematical model. Using this, we created a new, segregation-dependent model that describes the local thermal conductivity of polymer composites as a function of filler content with great accuracy.

## 1. Introduction

Filled polymers are widely used because they are cost-effective and have advantageous properties. Fillers can be spherical shaped, plates or fibers. They can be of many materials ranging from cheap minerals [1,2,3], such as chalk or talc, which reduce the price of the product, to high-quality fibrous reinforcement [1,4]. The properties of these materials are mostly determined by their macrostructure (shape, size, concentration, distribution and orientation), but their microstructure (morphological properties, the properties of the components, and their interaction) also affects their properties. Macrostructure properties can be influenced by the choice of filler and technological settings [5,6].

Fillers and reinforcement are widely used to increase the thermal conductivity of polymers because this is one of the most effective ways of producing thermally conductive polymer composites, which are more and more popular in industrial applications. Due to an increase in local temperature, the reliability and durability of electronic semiconductor devices can decrease. Metals and ceramics, with their high thermal conductivity, have been used to address this problem, but thermally conductive polymer composites can be used as well. They have many significant advantages: they are cheap, lightweight and easy to manufacture. More and more thermally conductive polymer structures are needed for the electronics industry, mainly because of the spread of electronic devices, for example flexible electronics, three-dimensional chip stacking, etc. [7,8,9].

Unfilled polymers have sensitive thermal conduction; they can be affected by chemical composition, bond strength, side group molecular weight, structure type, molecular density distribution, structural defects, processing parameters, temperature, and several other parameters [10,11]. The thermal conductivity of polymers is usually increased by adding good thermal conductivity fillers. These fillers can be carbon-based, ceramic-based or metal-based. The most commonly used carbon-based fillers are graphite, graphene, carbon nanotubes, carbon fibers and carbon black, but they all also significantly increase the electrical conductivity [10,12]. Their advantages are high thermal conductivity and low density. The metal powders most commonly used in the literature are aluminum, silver, copper and nickel powder. Metal powders increase both the thermal and electrical conductivity of the composites. However, if the amount of metal filler is increased in the polymer matrix, the density of the composite material increases considerably, and, in this way, a great advantage of polymer composites, low density (lightweight products), is lost. The great advantage of ceramics is that although they have good thermal conductivity, they are electrical insulators, therefore they are mostly used in the electronics industry as a heat absorbent. In the literature, the most commonly used fillers are beryllium oxide (BeO), aluminum nitride (AlN), boron nitride (BN) and silicon carbide (SiC). There are great differences in the properties of even the same fillers, due to the difference in their purity, crystallite structure, particle size and not least the method of thermal conductivity measurement. In this respect, some fillers (fibers and plates) are greatly anisotropic; they have better thermal conductivity along their main axis, or parallel to the surface of the plate [13]. As the filler content increases, the thermal conductivity of the material increases too, but if the filler content is too high, processability is impaired [8,14,15,16].

In the case of injection-molded products containing both filler and reinforcement, such as with thermally conductive polymers, the distribution of solid particles is often not homogeneous [17,18] either in the cross section of the part or along the flow path, which is called the segregation of the filler or reinforcement [1,19,20,21,22]. In addition to segregation, the orientation of the injection-molded product is also important. When the melt is flowing, the fibers are oriented due to the high shear, that is, they are ordered according to the flow conditions. This means that in the thin outer layer, the fibers are mostly parallel to the flow, while in the core part they are randomly oriented or perpendicular to it. This multi-layer structure can be controlled with the processing parameters and the geometry of the cavity and the gate [5,6,23].

Segregation was examined by Hegler and Mennig [5,6], among others. They used PA6 and SAN filled with glassed beads and glass fibers. They found that the larger the glass beads were, the more inhomogeneous their distribution was. Around the gate filler fraction was low, while away from the gate it was high. They also found that if short glass fibers were used, reinforcement content did not differ from the nominal value along the flow path. Papathanasiou and Ogadhoh [20,21] examined a PS matrix filled with glass beads. They used glass beads in five size ranges, from 40 μm to 500 μm. They found that segregation was negligible with smaller beads, but as size increased, segregation became more and more pronounced. They also showed that the distribution of the beads near the gate is considerably affected by the design and location of the gate, but further away from the gate, this effect is reduced, then disappears. Kovács [22] injection-molded flat 80 mm × 80 mm × 2 mm specimens from PA6, filled with 10 m%, 20 m%, 30 m% and 40 m% glass beads, and examined segregation in the samples. He measured filler distribution in the specimens in three locations in the flow direction and in three locations perpendicular to it. He found that filler distribution is even perpendicular to the flow direction but found significant differences parallel to the flow direction.

There are many papers in the literature focusing on thermally conductive polymer composites and on the segregation of fillers. We, however, have not found any publications that dealt with the effect of segregation on thermal conductivity and its modeling. Our goal is to investigate the segregation of fillers with the use of glass beads of different sizes, and make a descriptive model for the inhomogeneous distribution experienced. We also examine and model the inhomogeneous thermal conductivity caused by segregation in injection-molded samples.

## 2. Materials and Methods

### 2.1. Materials

H145 F homo-polypropylene (MOL Petrochemicals Co. Ltd., Tiszaújváros, Hungary) was used as a matrix material. The melt flow rate of this polypropylene is 29 g/10 min, and its tensile modulus and tensile strength at yield is 1.75 GPa and 39 MPa, respectively. We used glass beads (Cerablast GmbH & Co.KG, Löchgau, Germany) as a filler to investigate the segregation effect and to enhance the thermal conductivity of pure polypropylene.

### 2.2. The Preparation of the Samples

The preliminary microscopy examination of the glass beads showed that their size distribution is quite wide, therefore we separated them into fractions with a Cisa BA 200N electromagnetic and digital sieve shaker (CISA Cedaceria Industrial, Barcelona, Spain). We used the sieves with 75, 125 and 250 µm openings to make fractions between 0 µm and 75 µm, and between 125 µm and 250 µm. In these size ranges the effect of filler size on segregation will probably be noticeable. Before processing, we examined the size distribution of the glass beads with a Keyence VHX5000 (Keyence, Osaka, Japan) digital optical microscope.

We compounded the polypropylene matrix with 5 vol %, 10 vol % and 20 vol % glass beads using a Labtech LTE 25–30/C (Thailand) single-screw extruder. The proper amount of matrix material and filler was calculated with Equation (1) and Equation (2).

(1) $$m_{f}=\frac{m_{c}}{\frac{\phi_{m}\cdot \rho_{m}}{\phi_{f}\cdot \rho_{f}}+1}$$

(2) $$m_{m}=m_{c}-m_{f}$$

where *m_c_*, *m_f_* and *m_m_* (g) are the weight of the composite, the filler and the matrix material, *ϕ_f_* and *ϕ_m_* (vol %) are the volume fraction of the filler and matrix material, and finally *ρ_f_* (2.6 g/cm^3^) and *ρ_m_* (0.95 g/cm^3^) are the density of the filler and matrix material, respectively.

Traditionally, a twin-screw extruder has been used for compounding, but in our case, the glass beads are prone to breaking. We did not want to break them so we used a single-screw extruder and a rotational speed of 25 1/min and a melt temperature of 230 °C. Then we injection-molded flat specimens with a nominal size of 80 mm × 80 mm × 2 mm with an Arburg Allrounder (ARBURG Holding GmbH + Co. KG, Loßburg, Germany) 370 S 700-290 injection molding machine. Table 1 contains the main injection molding parameters.

### 2.3. The Investigation of Segregation

**Figure 1:**
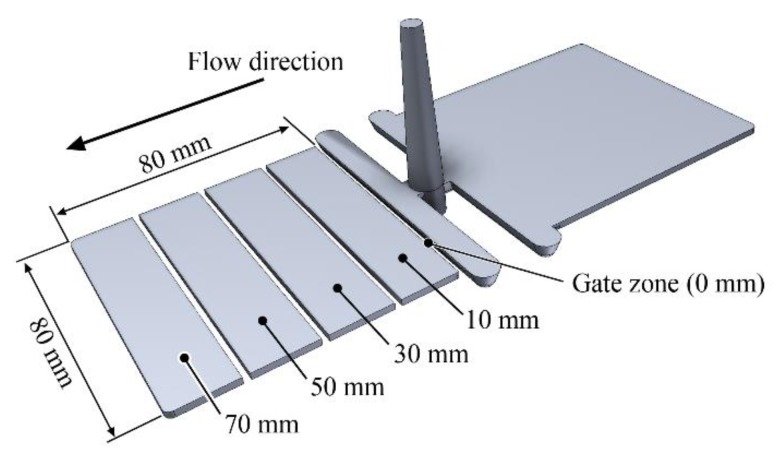
Preparation of the sample for the investigation of segregation.

In order to investigate segregation, we cut the injection-molded flat specimens into four equal 20 mm wide parts along the flow path (Figure 1). In this way, we were able to examine the amount of filler in four segments along the flow path.

We placed the specimens into a crucible whose mass we measured earlier (*m_c_*), then we measured the combined mass of the crucible and the specimen (*m_s+c_*). After this, we put the prepared samples in a Denkal (Kalória Hőtechnikai Kft., Budapest, Hungary) 6B furnace. According to method “A” of the ISO 3451-1 standard, we kept the sample at 600 °C for 4 hours, until the matrix material burned completely. Then we measured the combined weight of the crucible and the residual material (*m_a+c_*). The glass bead content (*ϕ_f_* [m%]) of the samples can be calculated with the following formula:

(3) $$\phi_{f}=\frac{m_{a+c}-m_{c}}{m_{s+c}-m_{c}}\cdot 100[\%]$$

The results were converted into volume fraction for further use. We also examined the fracture surfaces of the specimens with an electron microscope to learn about the distribution of the glass beads across the cross section. We took samples from the beginning and the end of the flow path, produced cryogenic fracture surfaces and examined them with a JEOL JSM 6380LA (Jeol Ltd., Tokyo, Japan) scanning electron microscope in BEC (back-scattered electron composition) mode.

### 2.4. The Measurement of Thermal Conductivity

**Figure 2:**
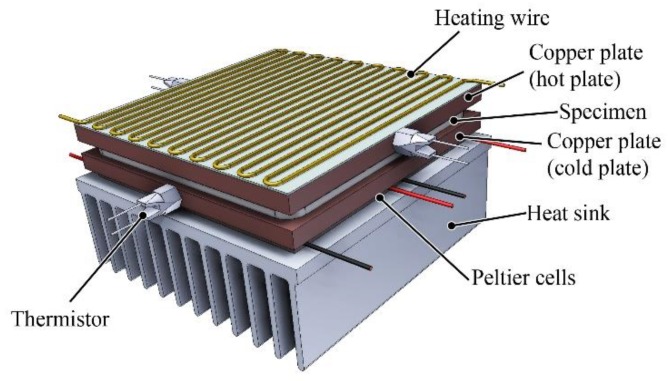
Assembly of the hot-plate measuring unit without thermal insulation.

The thermal conductivity was measured with a hot plate apparatus, which we developed earlier [24]. The cold plate of the apparatus was cooled by four Peltier cells. The hot plate was heated by a heating wire. The temperature was measured with two thermistors on each side (Figure 2). During the measurement process, a steady state condition in heat flow has to be achieved, and then the one-dimensional case of Fourier's law can be used:

(4) q(x,t)=−λ⋅∇T(x,t)

where *q* (W/m^2^) is the transmitted heat flux, λ (W/(mK)) is the thermal conductivity and *T* (K) is the temperature. The average test temperature was 55 °C.

## 3. Results and Discussion

### 3.1. Morphological Investigation

Before compounding, we analyzed the diameter of the glass beads with an optical microscope, separated into two different groups with the sieve shaker. We made the distribution diagrams using the diameters of a minimum of 700 glass beads in both fractions. This was crucial because we wanted to see if the two fractions are clearly separated from each other and to find the characteristic glass bead sizes of the fractions.

**Figure 3:**
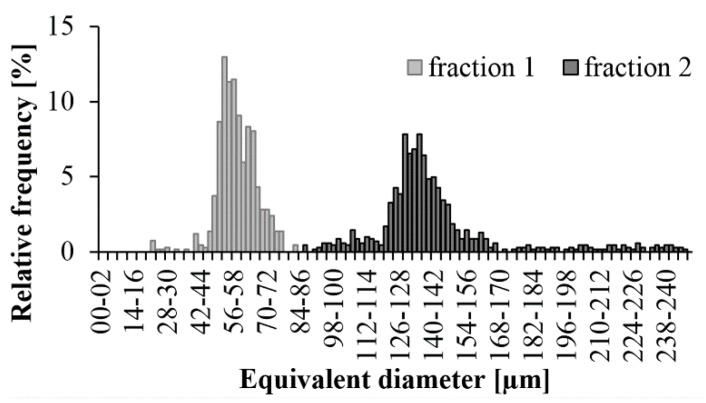
Glass bead size distribution after fractioning.

In both cases, we managed to create a relatively narrow size range (Figure 3). In the nominal range of 0–75 µm, we had beads between 45 µm and 75 µm, and the peak was at 55 µm. In the 125–250 µm range, the glass beads are characteristically between 120 µm and 165 µm. There were very few beads over 165 µm. Beads under 125 µm probably remained due to the short sieving time. This fraction is characterized by a peak of 135 µm.

**Figure 4:**
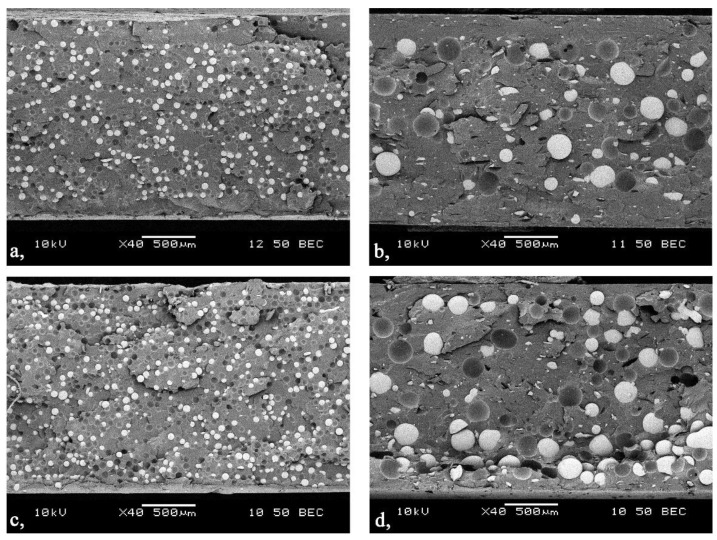
Distribution of the glass beads (20 vol %) in the injection-molded sample (45–75 µm: **a,** gate zone; **c,** end of the flow; 120–165 µm: **b,** gate zone; **d,** end of the flow).

With a scanning electron microscope we examined the distribution of the glass beads across the cross section of the injection-molded samples containing 20 vol % glass beads (GB). The purpose of the micrographs was to show whether the flow of the melt affects segregation through the cross section. Figure 4 shows that the smaller beads are distributed almost uniformly through the cross section, both at the beginning and the end of the flow path. At the same time, near the gate, on the surface of the sample, a 100–150 µm thick layer formed, which had less filler in it. At end of the flow path, this layer disappeared. When larger glass beads were used, the layer with fewer beads at the beginning of the flow path was thicker, about 200–250 µm, and the larger beads were in the middle of the cross section. At the end of the flow path, this phenomenon was not perceptible—larger particles appeared near the surface, too. This proves that the size of filler particles affects segregation through the cross section.

### 3.2. Model for the Segregation of Glass Beads

**Figure 5:**
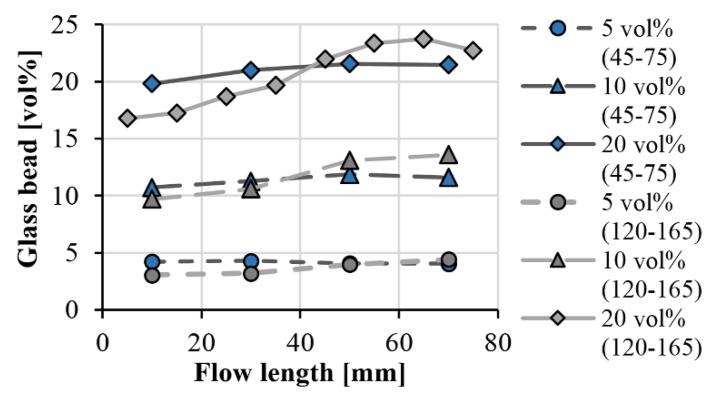
Glass bead concentration along the flow length as a function of bead size.

We measured the amount of filler at four segments at different distances from the gate (from where the melt enters into the cavity). In the case of the compound containing 20 vol % 120–165 µm glass beads, we used eight measurement points for greater accuracy. Figure 5 shows the measurement results. It indicates that in the case of smaller (typically 55 µm) particles, we did not experience considerable segregation; even at 20 vol % filler content, the difference between the beginning and end of the flow path was only 1.6 vol %. Using bigger (typically 135 µm) particles, we found noticeable segregation. The segregation increases as the filler content increases: at filler contents of 5 vol %, 10 vol % and 20 vol %, the difference between the beginning and the end of the flow path was 1.4 vol %, 3.9 vol % and 6.0 vol %, respectively. The inaccuracy of the measurement of the glass bead concentration was under 1.5% for each case.

Glass bead content along the flow path in injection-molded products showed a saturation character with an inflexion point, which can be described with a logistic function well. The general form of the function is:

(5) $$\phi (l)=c_{1}+\frac{c_{2}}{1+e^{-s\cdot (l-l_{i})}}$$

where *ϕ* (*l*) (vol %) is the local filler content, *l* (mm) is the measurement position in the sample (flow length), measured from the gate, *c*_1_ is the minimum of the curve, *c*_2_ is the range of the function, l (mm) is a variable parameter giving the current position along the flow path, *l*_i_ (mm) is the inflexion point of the curve, and *s* (1/mm) is the slope of the transient section.

In our case, the *c*_1_ parameter of Equation (5) is the glass bead content at the beginning of the flow path (*ϕ*_0_ (vol %)) (Equation (6)).

(6) $$c_{1}=\phi_{0}$$

Parameter *c*_2_ is the difference between glass bead content at the end of the flow path (*ϕ_end_* (vol %)) and at the beginning of the flow path (Equation (7)).

(7) $$c_{2}=\phi_{end}-\phi_{0}$$

Combining Equations (5), (6) and (7), we get Equation (8), which describes local bead content and segregation.

(8) $$\phi (l)=\phi_{0}+\left[\frac{\phi_{end}-\phi_{0}}{1+e^{-s\cdot (l-l_{i})}}\right]$$

**Figure 6:**
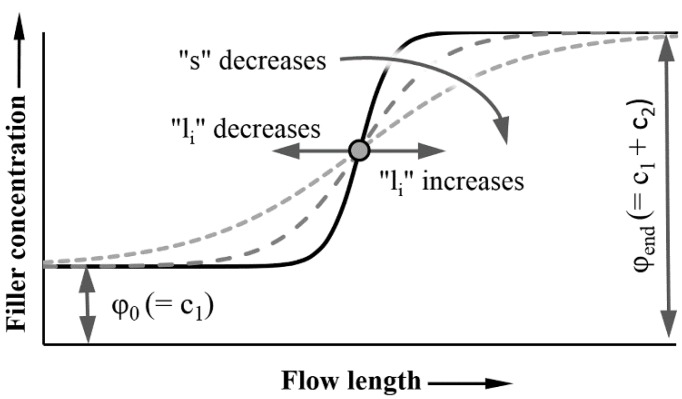
Interpretation of the factor of the logistic curve used to describe the segregation along the flow length.

Figure 6 helps interpret the parameters of Equation (8).

**Figure 7:**
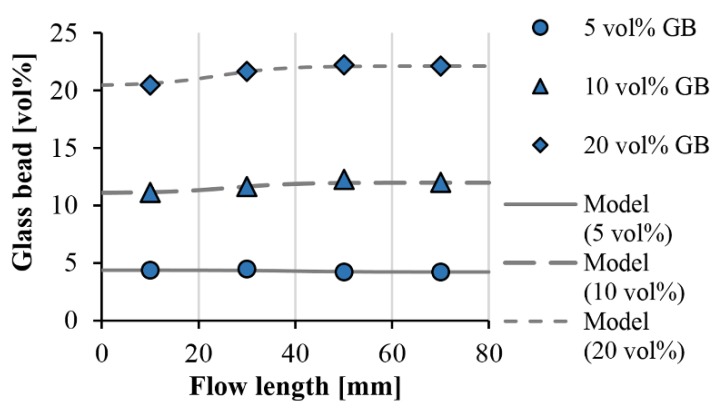
Segregation of the glass beads along the flow path (measurement results and the fitted model; GB size: 45–75 µm).

**Figure 8:**
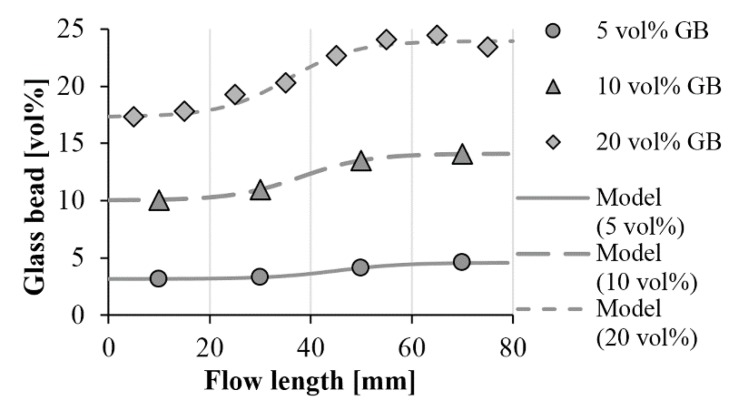
Segregation of the glass beads along the flow path (measurement results and fitted model; GB size: 120–165 µm).

We fitted the formula on the measured points. Figure 7 and Figure 8 show the measurement points and the fitted curves. With both glass bead sizes, the function is highly accurate, the RMSE (root-mean-square error) for the individual data series does not exceed 0.4 vol %.

Table 2 and Table 3 show the fitting parameters (*s* and *l_i_*) for the compounds containing the 45–75 µm and 120–165 µm glass beads. The curves fit well when parameter *s*, which describes the character of the transition, is 0.15. The value of this parameter is probably determined by injection molding process parameters and the geometry of the product. In addition, as filler content increases, the inflexion point of the function is shifted to shorter flow paths. One possible reason is that increasing filler content increases the possibility of segregation—when concentration is higher, the flow is more likely to carry along the glass beads.

### 3.3. Thermal Conductivity of the Samples

**Figure 9:**
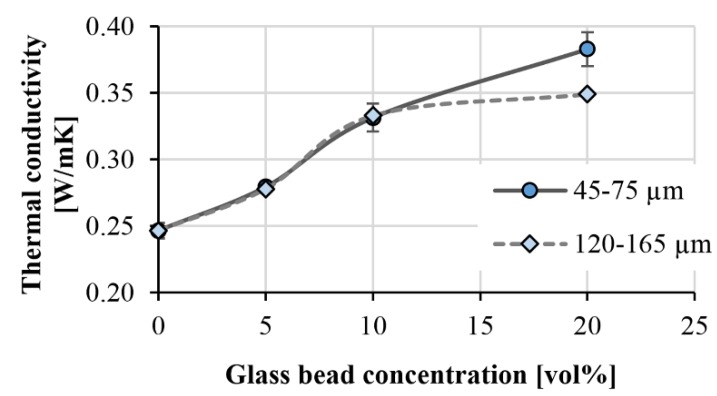
Thermal conductivity of filled polypropylenes.

We measured the thermal conductivities of the injection-molded samples with the hot-plate apparatus we presented earlier. The device can only test samples with an area of 80 mm × 80 mm, therefore we could not measure the local thermal conductivities. Figure 9 shows the thermal conductivities of the different PP matrix compounds. Between 0 vol % and 10 vol % glass bead content, the thermal conductivity was not significantly affected by the diameter of the beads. We experienced an increase of 13.3% and 34.4% in the case of 5 vol % and 10 vol %, respectively, compared to unfilled PP. At 20 vol %, the size of the beads had a significant effect. The larger beads resulted in a 13.8% lower thermal conductivity than the 45–75 µm beads. This is probably due to segregation, that is, inhomogeneous distribution.

**Figure 10:**
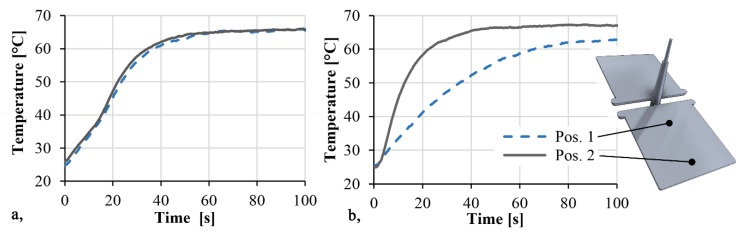
Heating curves of the injection-molded 20 vol % GB filled samples (**a,** GB size: 45–75 µm; **b,** 120–165 µm).

We proved the effect of inhomogeneous distribution on thermal conductivity with a FLIR A325sc (FLIR Systems, Wilsonville, OR, USA) thermal imaging camera. We placed the 20 vol % GB samples from the different series on a 70 °C metal plate and measured the surface temperatures of the samples as a function of time near the gate and at the end of the flow path, with a thermal imaging camera (Figure 10). The emission coefficient was set to 0.95. In the case of small beads, where segregation is negligible, the temperature increased at both ends of the product at the same rate. The average heating rate between 30 °C and 50 °C was 1.1 °C/s. In the case of 120–165 µm beads, the difference was considerable. We measured an average heating rate of 0.7 °C/s at the beginning of the flow path, while at the end, it was 2.3 °C/s, which is due to segregation.

### 3.4. Developing a Model for Segregation

We can conclude from the thermal conductivity of the samples and the inhomogeneous filler distribution that the thermal conductivity within the sample changes along the flow path. In order to show the effect of segregation on the thermal conductivity along the flow path, we further developed the semi-empirical model we had created earlier [25]. The original model can be written as (9), which describes the thermal conductivity of particle-filled polymer composites as a function of filler content.

(9) $$\lambda_{c}={\lambda^{\prime }}_{m}\cdot \left[1-{\left(\frac{\phi }{\phi_{\max}}\right)}^{C}\right]+{\lambda^{\prime }}_{f}\cdot {\left(\frac{\phi }{\phi_{\max}}\right)}^{C};\;\left(0\leq \phi \leq \phi_{\max}\right)$$

where *λ_c_* is the thermal conductivity of the composite, *λ*’*_m_* and *λ*’*_f_* are the effective thermal conductivities of the matrix and the filler, *ϕ* and *ϕ*_max_ are the filler content and the maximum achievable filler content, and *C* is a constant describing the conductive chain formation capability and shape factor of the material. *λ*’*_m_* and, *λ*’*_f_* can be determined by direct measurements, or can be calculated from values in the literature.

In Equation (9), if the segregation model (8) is substituted in the place of *ϕ*, we get Equation (10), which describes the thermal conductivity inhomogeneity in the sample due to segregation, when the value of parameter C is known.

(10) $$\lambda_{c}(l)={\lambda^{\prime }}_{m}\cdot \left[1-{\left(\frac{\phi (l)}{\phi_{\max}}\right)}^{C}\right]+{\lambda^{\prime }}_{f}\cdot {\left(\frac{\phi (l)}{\phi_{\max}}\right)}^{C};\;\left(0\leq \phi (l)\leq \phi_{\max}\right)$$

First we fitted Equation (9) to the thermal conductivities of the compounds containing smaller (0–75 µm) glass beads. Because, in this case, there was no significant segregation, these specimens characterize the thermal conductivity of the homogeneous sample. In our model, we used the following parameters:

- *λ*’_*m*_ = 0.25 W/mK
- *λ*’_*f*_ = 0.8 W/mK [26]
- *ϕ*_max_ = 63%
- *C* = 1.12

**Figure 11:**
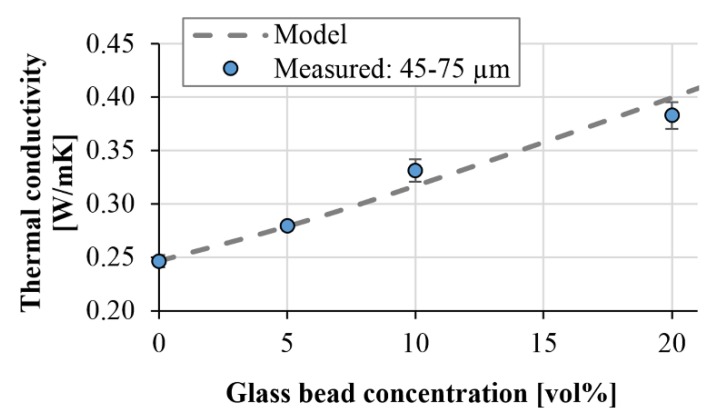
Comparison of the measured and modeled thermal conductivity (glass bead size: 45–75 µm).

To determine maximum filler content (*ϕ*_max_), we put the glass beads in a container of given volume, then we measured the mass of the beads and calculated their (bulk density). After this, we divided this with the actual density of the glass beads (2.5 g/cm^3^) in the datasheet, and got *ϕ*_max_ (0.63). Using the parameters, we fitted the formula on the measured values. We obtained the best fitting with *C* = 1.12 (Figure 11).

**Figure 12:**
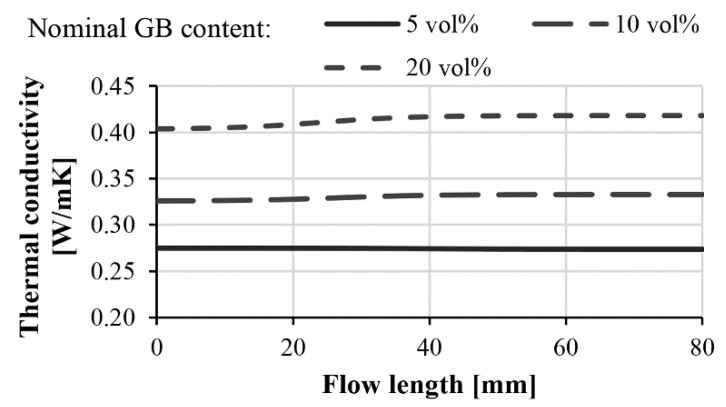
Calculated thermal conductivity of glass bead (45–75 µm)-filled polypropylene along the flow length.

With parameter C and Equations (8) and (10), the local thermal conductivities in injection-molded composite products can be modeled as a function of the flow path and filler content. Figure 12 shows the local thermal conductivities of injection-molded samples filled with 45–75 µm diameter glass beads. In the case of such small beads, no significant difference can be detected between the thermal conductivities at the beginning and at the end of the flow path.

**Figure 13:**
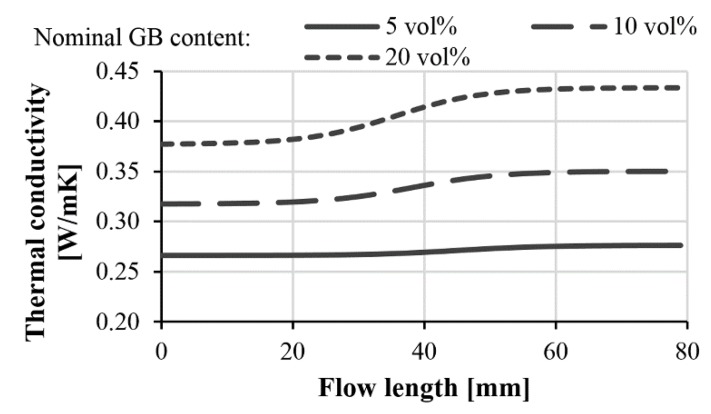
Calculated thermal conductivity of glass bead (120–165 µm)-filled polypropylene along the flow length.

In injection-molded composites containing glass beads in the range 120–165 µm, segregation had a considerable effect on the distribution of the filler. This inhomogeneous distribution affected thermal conductivities too (Figure 13). A nominal GB content of 5 vol %, 10 vol % and 20 vol % caused a difference of 4%, 10% and 15% in thermal conductivity between the beginning and end of the flow path, respectively. At the highest investigated glass bead content, our model predicted the thermal conductivity at the beginning of the flow path to be 0.37 W/mK, and 0.43 W/mK at the end of the flow path. If particles with a higher thermal conductivity (but with the same size and aspect ratio) are used, the difference can increase considerably.

## 4. Conclusions

We modeled segregation in injection-molded samples and its effect on local thermal conductivity. We compounded 5 vol %, 10 vol % and 20 vol % glass beads of different sizes (45–75 µm and 120–165 µm) with polypropylene homopolymers, and then injection-molded samples from the compounds. We determined local glass bead content by calcination tests, and showed that segregation is negligible in the case of the smaller beads but in the case of the larger ones, it is considerable. In the case of 20 vol % glass beads with a characteristic size of 135 µm, the difference between the beginning and the end of the flow path was 6 vol % (~9.1 m%). We created a formula, which describes local filler content in the injection-molded samples with great accuracy. The s and li fitting parameters of the function show the slope and the inflexion point of the transition of the model. When parameter s was 0.15, the root-mean-square error did not exceed 0.4 vol % in any data series. We also showed that as filler content increases, the inflexion point of the function is shifted towards shorter flow paths. This is caused by the saturation of the matrix with glass beads at the end of the flow path.

We combined the segregation model with the formula we had worked out earlier, which gives the thermal conductivity of polymer composites as a function of filler content. We combined the two formulas to model and predict the inhomogeneities in local thermal conductivity caused by segregation. There was no significant difference in the local thermal conductivities of samples containing glass beads in the size range 45–75 µm. When the larger glass beads were used, however, we experienced a larger difference between the thermal conductivities at the beginning and at the end of the flow path. At a nominal filler content of 5 vol %, 10 vol % and 20 vol %, these differences were 4%, 10% and 15%, respectively. We proved the effect of segregation on local thermal conductivity with thermal camera images.

## Figures and Tables

**Table 1:** Injection molding parameters.

Parameter	Unit	Value
Volume	[cm^3^]	44
Switch-over volume	[cm^3^]	8
Plasticizing speed	[m/min]	20
Injection rate	[cm^3^/s]	80
Holding pressure	[bar]	350
Clamping force	[tonne]	70
Cooling time	[s]	25
Zone temperatures	[°C]	230; 225; 220; 215; 210
Mold temperature	[°C]	40

**Table 2:** Parameters of the segregation model and the RMSE of fitting for 45–75 µm glass beads.

Nominal Filler Concentration	Unit	5 vol %	10 vol %	20 vol %
*ϕ*_0_	[vol %]	4.4	11.1	20.5
*ϕ_end_*	[vol %]	4.2	12	22.1
*s*	[1/mm]	0.15	0.15	0.15
*l_i_*	[mm]	40	26.6	24
*RMSE*	[vol %]	0.05	0.15	0.12

**Table 3:** Parameters of the segregation model and the RMSE of fitting for 120–165 µm glass beads.

Nominal Filler Concentration	Unit	5 vol %	10 vol %	20 vol %
*ϕ*_0_	[vol %]	3.2	10	17.3
*ϕ_end_*	[vol %]	4.6	14.1	23.9
*s*	[1/mm]	0.15	0.15	0.15
*l_i_*	[mm]	45	38	35.5
*RMSE*	[vol %]	0.02	0.04	0.4
